# Dietary Bacteriophage Administration Alleviates Enterotoxigenic *Escherichia coli*-Induced Diarrhea and Intestinal Impairment through Regulating Intestinal Inflammation and Gut Microbiota in a Newly Weaned Mouse Model

**DOI:** 10.3390/ijms251910736

**Published:** 2024-10-05

**Authors:** Chao Dong, Yan Chen, Minfeng Ding, Yi Liu, Xingping Chen, Yuyong He, Tiande Zou, Jun Chen, Jinming You

**Affiliations:** Jiangxi Province Key Laboratory of Animal Nutrition and Feed, Jiangxi Province Key Innovation Center of Integration in Production and Education for High-Quality and Safe Livestock and Poultry, Jiangxi Agricultural University, Nanchang 330045, China

**Keywords:** bacteriophage, diarrhea, enterotoxigenic *Escherichia coli*, gut microbiota, intestine, newly weaned mice

## Abstract

This study aimed to investigate the effects of dietary bacteriophage administration on diarrhea and intestinal impairment induced by enterotoxigenic *Escherichia coli* (ETEC) in a newly weaned mouse model. Forty-four newly weaned C57BL/6 mice were divided into four treatment groups, where they were provided either the control diet or the bacteriophage-supplemented diet, with or without ETEC infection. The results show that the bacteriophage administration resulted in increased body weight, decreased diarrhea score, and improved jejunal histopathology in ETEC-infected mice. The bacteriophage administration enhanced the intestinal barrier function of the ETEC-infected mice, as indicated by the reduced serum DAO level and the increased expression of Claudin-1, Occludin, and ZO-1 at both the mRNA and protein levels in the jejunum. Also, the bacteriophage administration resulted in a decrease in serum TNF-α and IL-1β levels, a down-regulation of *TNF-α* and *IL-6* mRNA levels in the jejunum, and the inhibition of jejunal TLR-4/NF-κB pathway activation induced by ETEC infection. Moreover, the bacteriophage administration increased the levels of acetic acid, propionic acid, butyric acid, and total short-chain fatty acids in the caecum content. The bacteriophage administration increased the Shannon index, increased the abundance of *Bacteroidota* and *Muribaculaceae*, and decreased the abundance of *Verrucomicrobiota* and *Akkermansiaceae* in the colon contents of the ETEC-infected mice. Spearman’s correlation analysis indicates that the protective effects of bacteriophage on ETEC-induced intestinal impairment, inflammation, and intestinal barrier function are associated with regulating the abundance of *Bacteroidota* and *Muribaculaceae* in the colon contents of mice. Collectively, bacteriophage administration alleviates ETEC-induced diarrhea and intestinal impairment through regulating intestinal inflammation and gut microbiota in newly weaned mice.

## 1. Introduction

Enterotoxigenic *Escherichia coli* (ETEC) is the prevailing form of colibacillosis in human infants and young animals, as well as a substantial etiological factor in instances of diarrhea of travelers and children in underprivileged areas [1,2]. Notably, ETEC K88 is the predominant serotype of ETEC, capable of causing intestinal infections and diarrhea in neonates and juvenile animals [3,4]. Furthermore, infection with ETEC K88 can result in illnesses characterized by diarrhea, significant weight loss, and even mortality [5,6]. Consequently, ETEC K88 represents a substantial threat to the health of human infants and young animals within the livestock industry.

Antibiotics are typically prioritized as the initial treatment for ETEC infection. However, the widespread use of antibiotics has resulted in the emergence of multidrug-resistant strains, which are now becoming more prevalent in both animal and human populations [7]. The escalating rates of antimicrobial resistance have reinvigorated research on bacteriophages, the naturally occurring predators of bacteria that were first discovered more than a century ago [8]. Bacteriophages are reported as a possible alternative for effectively controlling pathogenic bacteria [9,10]. In our previous study, it has been demonstrated that bacteriophage, as a substitute to antibiotics, enhanced growth performance and intestinal health in a newly weaned piglet model through the regulation of gut inflammation, barrier function, and microbiota [11]. Also, under the ETEC challenge condition, dietary or oral administration with bacteriophage was found to significantly alleviate ETEC infection symptoms in weaned piglets [12,13]. Furthermore, in the IPEC-J2 cell model, it has been reported that bacteriophage has the ability to alleviate barrier dysfunction and inflammation induced by ETEC [14]. The safety of administering bacteriophage has been reported in human studies that involve either children or adults [15,16,17,18,19]. Therefore, bacteriophages represent a promising alternative with the potential to effectively and safely alleviate intestinal infection and inflammatory responses caused by pathogenic bacteria.

However, there is presently limited data available regarding the protective effects of bacteriophage administration on diarrhea and intestinal impairment induced by ETEC in a newly weaned mouse model, as well as its potential underlying mechanism. The use of weaned mice is commonly employed in the establishment of the ETEC infection model for studies involving pediatric or juvenile animals. This model is widely employed to investigate innovative supplements aimed at alleviating intestinal diseases and injuries caused by ETEC infection [3,4,20]. Therefore, a model using an ETEC infection in newly weaned mice was employed to investigate the hypothesis that the administration of bacteriophage could effectively mitigate the negative consequences of ETEC-induced diarrhea and intestinal injury in newly weaned human babies or animals in the livestock industry.

## 2. Results

### 2.1. Body Weight and Diarrhea Score

**Figure 1:**
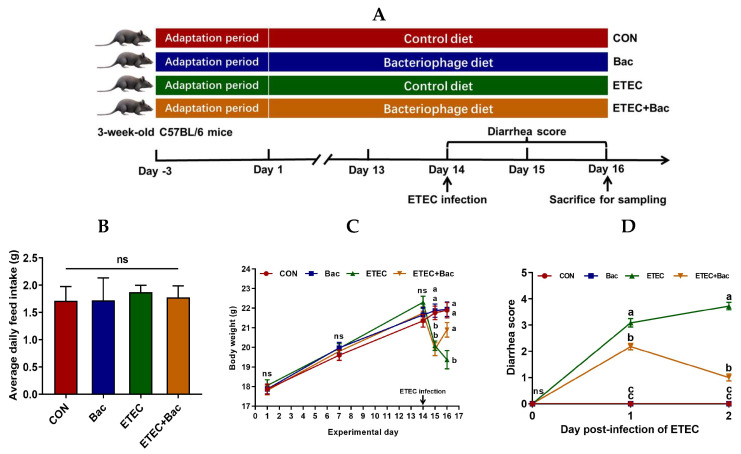
Experimental design and the effects of the bacteriophage administration on the body weight (BW) and diarrhea score in the ETEC-infected weaned mice. (**A**) Study protocol. (**B**) Average daily feed intake (ADFI). (**C**) BW. (**D**) Diarrhea score. CON (control diet); Bac (bacteriophage diet); ETEC (control diet with ETEC infection); ETEC + Bac (bacteriophage diet with ETEC infection). Data are expressed as mean ± SEM (*n* = 11). The bars represented by distinct letters signify significant statistical differences among the treatment groups (*p* < 0.05). The abbreviation ns denotes non-significant differences among the treatment groups (*p* > 0.05).

As shown in Figure 1, no statistical difference was observed in the body weight of the mice in the treatment groups prior to ETEC infection (*p* > 0.05). However, there was a decrease in body weight and an increase in the diarrhea score observed in the mice infected with ETEC at 24 h post-infection (*p* < 0.05). Importantly, compared with the ETEC group, the mice from the ETEC + Bac group exhibited increased body weight and decreased diarrhea score at 48 h post-infection of ETEC (*p* < 0.05). Herein, all the mice were sacrificed at 48 h post-infection to facilitate blood- and intestine-related sample collection and the subsequent laboratory analysis.

### 2.2. Intestinal Histomorphology

**Figure 2:**
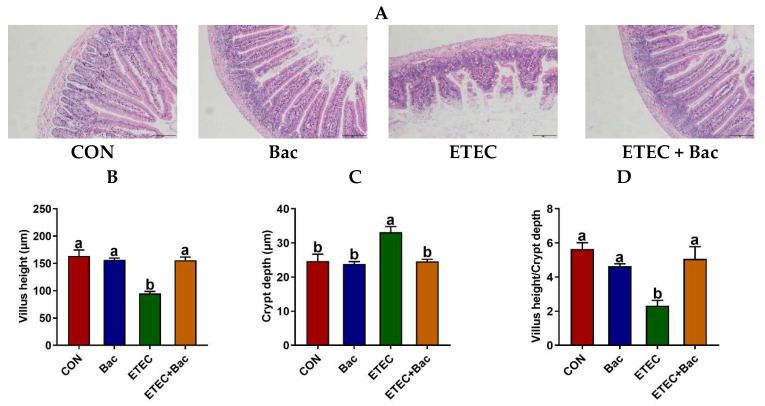
The histomorphology of the jejunum in the mice in response to the experimental treatments. (**A**) Representative hematoxylin and eosin staining images with a scale bar of 50 μm. (**B**) Villus height (VH). (**C**) Crypt depth (CD). (**D**) VH/CD. CON (control diet); Bac (bacteriophage diet); ETEC (control diet with ETEC infection); ETEC + Bac (bacteriophage diet with ETEC infection). Data are expressed as mean ± SEM (*n* = 11). The bars represented by distinct letters signify significant statistical differences among the treatment groups (*p* < 0.05).

Compared to the control (CON) group, the ETEC-infected mice exhibited impaired jejunal histopathology, as evidenced by an elevation in crypt depth (CD) and a reduction in villus height (VH) and VH/CD (*p* < 0.05) (Figure 2). However, the bacteriophage administration effectively alleviated the deleterious consequences of the ETEC infection on jejunal histopathology in the mice, as suggested by the decreased CD and the increased VH and VH/CD in the mice from the ETEC + Bac group vs. ETEC group (*p* < 0.05).

### 2.3. Intestinal Barrier Function

**Figure 3:**
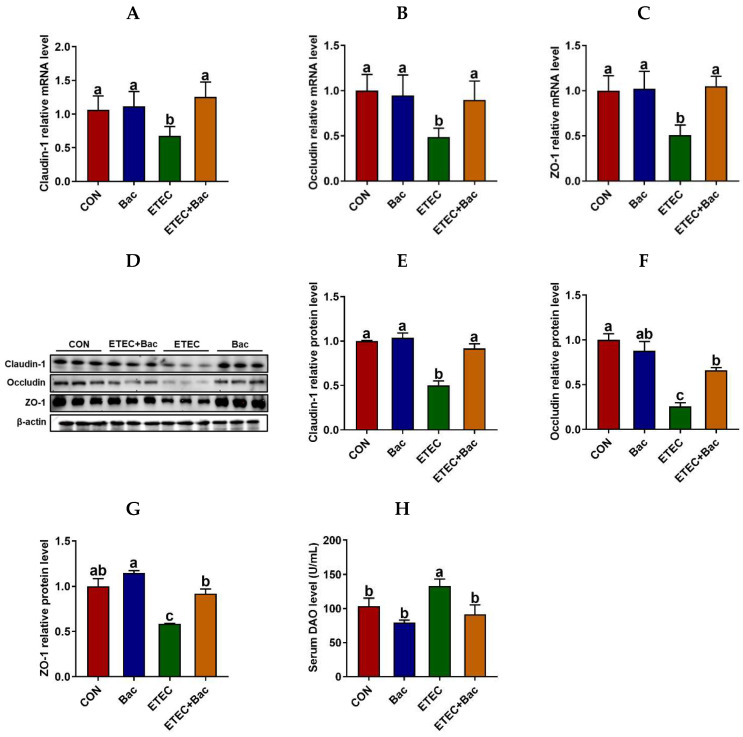
Intestinal barrier function in the mice in response to the experimental treatments. (**A**) Jejunal *Claudin-1* mRNA abundance. (**B**) Jejunal *Occludin* mRNA abundance. (**C**) Jejunal *ZO-1* mRNA abundance. (**D**) Western blotting bands. (**E**) Jejunal Claudin-1 protein abundance. (**F**) Jejunal Occludin protein abundance. (**G**) Jejunal ZO-1 protein abundance. (**H**) Serum diamine oxidase (DAO) level. CON (control diet); Bac (bacteriophage diet); ETEC (control diet with ETEC infection); ETEC + Bac (bacteriophage diet with ETEC infection). Data are expressed as mean ± SEM. There are 11 replicates for the mRNA expression levels of tight junction proteins and the serum DAO level. Meanwhile, there are 3 replicates for the protein abundances of tight junction proteins. The bars represented by distinct letters signify significant statistical differences among the treatment groups (*p* < 0.05).

As depicted in Figure 3, compared with the CON group, the mice in the ETEC group exhibited impaired intestinal barrier function, as evidenced by an increase in the serum diamine oxidase (DAO) level (*p* < 0.05). Furthermore, the mRNA and protein expression abundances of Claudin-1, Occludin, and ZO-1 were observed to be down-regulated in the jejunum mucosa of the mice from the ETEC group compared to the CON group (*p* < 0.05). Importantly, the bacteriophage administration enhanced the intestinal barrier function of the ETEC-infected mice. This is evident from the reduction in the serum DAO level and the increased mRNA and protein expression levels of Claudin-1, Occludin, and ZO-1 in the jejunum mucosa of the mice belonging to the ETEC + Bac group compared to those in the ETEC group (*p* < 0.05).

### 2.4. Inflammatory Response

**Figure 4:**
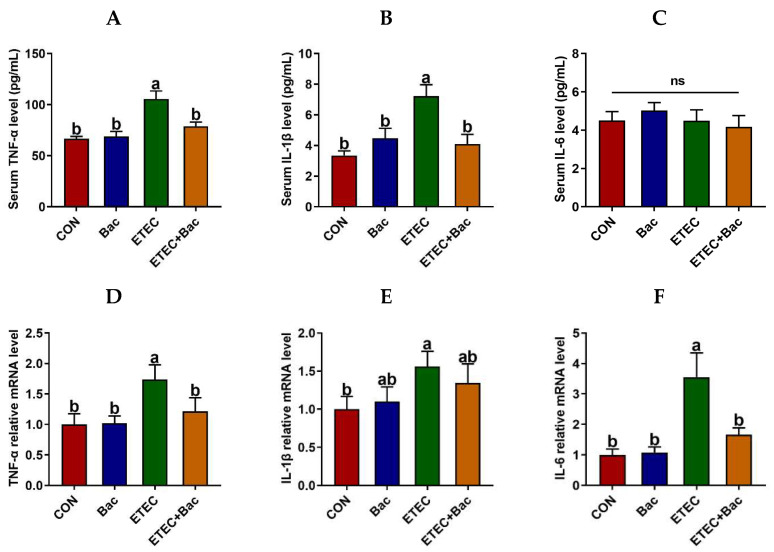
Inflammatory response in the mice in response to the experimental treatments. (**A**) Serum TNF-α level. (**B**) Serum IL-1β level. (**C**) Serum IL-6 level. (**D**) Jejunal *TNF-α* mRNA level. (**E**) Jejunal *IL-1β* mRNA level. (**F**) Jejunal *IL-6* mRNA level. CON (control diet); Bac (bacteriophage diet); ETEC (control diet with ETEC infection); ETEC + Bac (bacteriophage diet with ETEC infection). Data are expressed as mean ± SEM (*n* = 11). The bars represented by distinct letters signify significant statistical differences among the treatment groups (*p* < 0.05). The abbreviation ns denotes non-significant differences among the treatment groups (*p* > 0.05).

Figure 4 displays the levels of inflammatory factors and immunoglobulins in serum in the mice in response to the experimental treatments. The IL-6 level in the serum of the mice was unaffected by the administered treatments (*p* > 0.05). The serum levels of TNF-α and IL-1β were increased in the mice from the ETEC group vs. the CON group (*p* < 0.05). However, the serum levels of TNF-α and IL-1β were decreased in the mice from the ETEC + Bac group vs. the ETEC group (*p* < 0.05).

**Figure 5:**
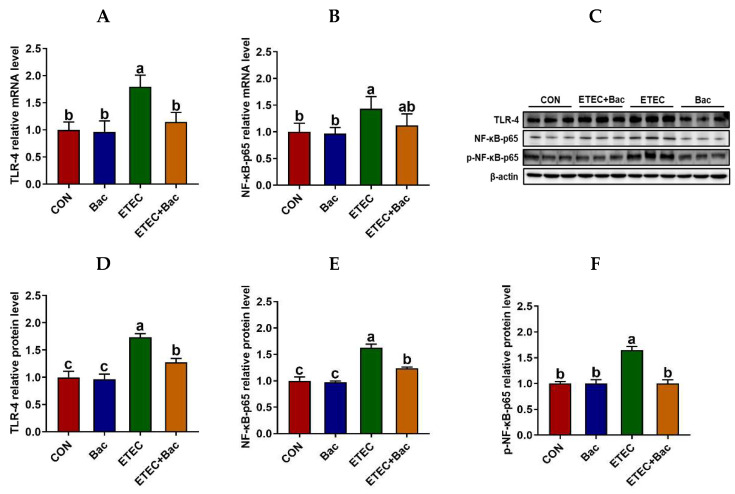
The TLR4/NF-κB pathway in the jejunum in the mice in response to the experimental treatments. (**A**) Jejunal *TLR-4* mRNA level. (**B**) Jejunal *NF-κB-p65* mRNA level. (**C**) Western blotting bands. (**D**) Jejunal TLR-4 protein level. (**E**) Jejunal NF-κB-p65 protein level. (**F**) Jejunal p-NF-κB-p65 protein level. CON (control diet); Bac (bacteriophage diet); ETEC (control diet with ETEC infection); ETEC + Bac (bacteriophage diet with ETEC infection). Data are expressed as mean ± SEM. There are 11 replicates for those parameters except for 3 replicates for Western blotting results. There are 11 replicates for the mRNA expression levels of *TLR-4* and *NF-κB-p65*. Meanwhile, there are 3 replicates for the protein expression levels of TLR-4, NF-κB-p65, and p-NF-κB-p65. The bars represented by distinct letters signify significant statistical differences among the treatment groups (*p* < 0.05).

We further evaluated the inflammatory response in the jejunum mucosa in the mice in response to the experimental treatments (Figure 5). In comparison to the CON group, the mice from the ETEC group exhibited up-regulated mRNA levels of *TLR-4* and *NF-κB-p65*, along with up-regulated protein levels of TLR-4, NF-κB-p65, and p-NF-κB-p65 in the jejunum mucosa (*p* < 0.05). Moreover, the mRNA levels of *TNF-α* and *IL-6* were up-regulated in the jejunum mucosa of the mice from the ETEC group vs. the CON group (*p* < 0.05). However, compared to the ETEC group, the mice from the ETEC + Bac group exhibited the down-regulation of mRNA levels for *TLR-4* and *NF-κB-p65*, as well as down-regulated protein levels for TLR-4, NF-κB-p65, and p-NF-κB-p65 (*p* < 0.05). Additionally, the mRNA levels of *TNF-α* and *IL-6* were observed to be down-regulated in the jejunum mucosa of the mice from the ETEC + Bac group as compared to the ETEC group (*p* < 0.05).

### 2.5. Short-Chain Fatty Acid (SCFA) Levels

**Figure 6:**
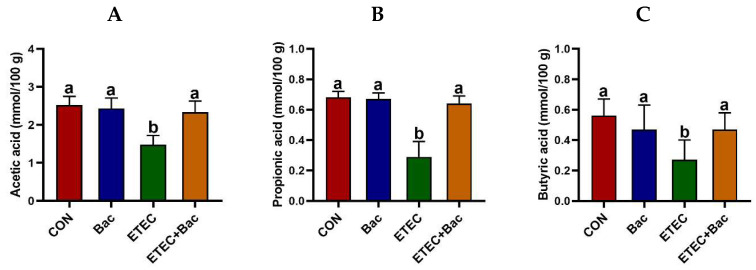

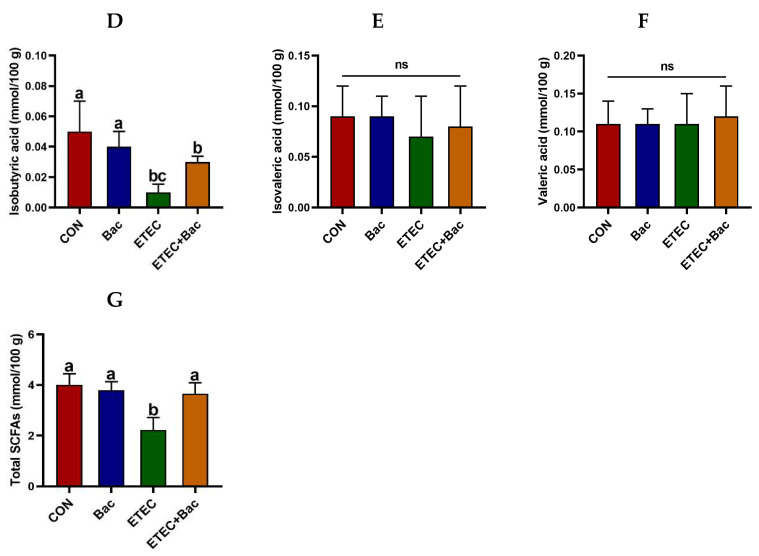
The content of short-chain fatty acids (SCFAs) in the caecum contents in the mice in response to the experimental treatments. (**A**) Acetic acid level. (**B**) Propionic acid level. (**C**) Butyric acid level. (**D**) Isobutyric acid level. (**E**) Isovaleric acid level. (**F**) Valeric acid level. (**G**) Total SCFAs level. CON (control diet); Bac (bacteriophage diet); ETEC (control diet with ETEC infection); ETEC + Bac (bacteriophage diet with ETEC infection). Data are expressed as mean ± SEM (*n* = 11) The bars represented by distinct letters signify significant statistical differences among the treatment groups (*p* < 0.05). The abbreviation ns denotes non-significant differences among the treatment groups (*p* > 0.05).

As depicted in Figure 6, the group of mice with ETEC infection showed a reduction in the levels of acetic acid, propionic acid, butyric acid, isobutyric acid, and total SCFAs in the contents of the caecum when compared to the CON group (*p* < 0.05). However, the levels of acetic acid, propionic acid, butyric acid, and total SCFAs in the caecum content were elevated in the mice from the ETEC + Bac group vs. the ETEC group (*p* < 0.05).

### 2.6. Diversity of Gut Microbiota

**Figure 7:**
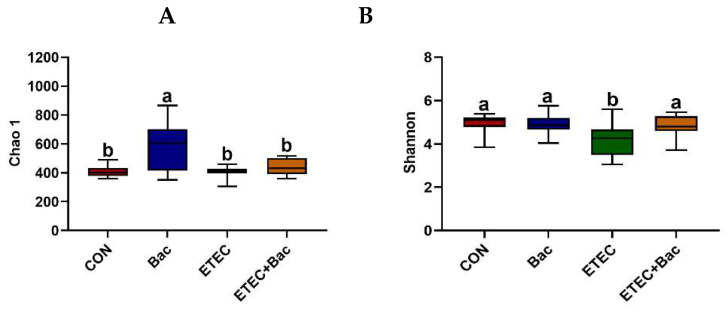

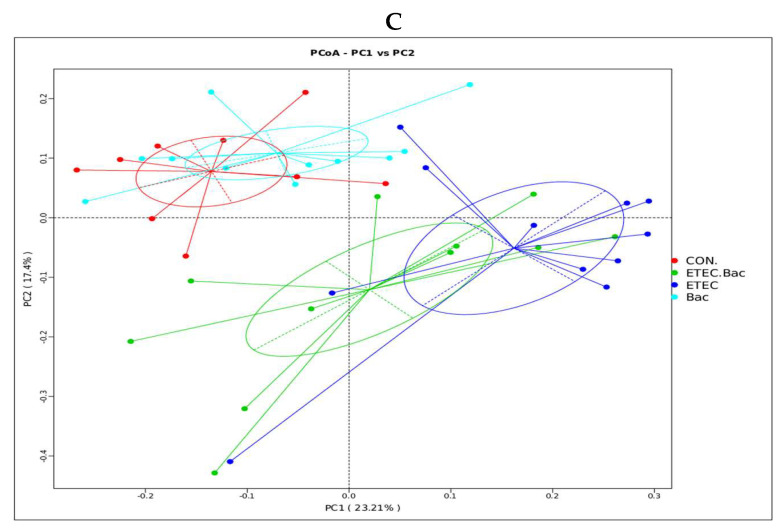
Microbiota diversity in the colon contents in the mice in response to the experimental treatments. (**A**) Chao 1. (**B**) Shannon. (**C**) β-diversity. CON (control diet); Bac (bacteriophage diet); ETEC (control diet with ETEC infection); ETEC + Bac or ETEC.Bac (bacteriophage diet with ETEC infection). *n* = 9 or 11. No colonic contents were available for sample collection when the mice were sampled, which resulted in *n* = 9 for the control group. The bars represented by distinct letters signify significant statistical differences among the treatment groups (*p* < 0.05).

Compared with the CON group, the mice from the ETEC group had decreased Shannon index in the colon contents (*p* < 0.05, Figure 7). However, the mice from the ETEC + Bac group had increased Shannon index in the colon contents compared to those from the ETEC group (*p* < 0.05). Regarding the β-diversity, statistical differences in significance were observed across the four treatment groups (*p* < 0.05).

### 2.7. Composition of Gut Microbiota

**Figure 8:**
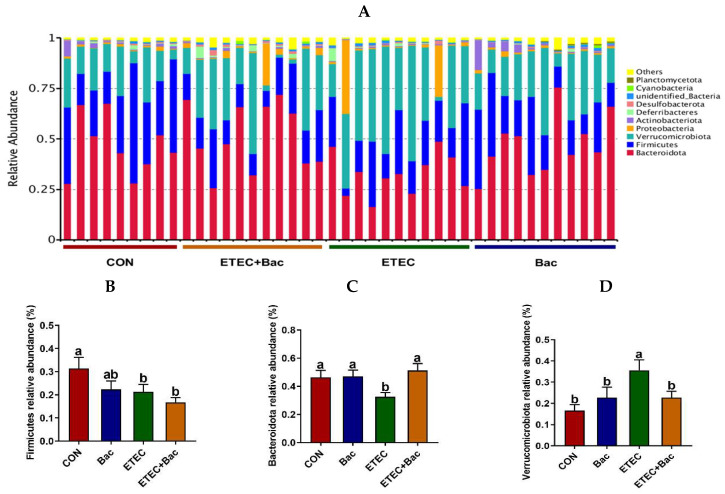
Microbiota composition at the phylum level (top 10) in the colon contents in the mice in response to the experimental treatments. (**A**) Microbiota composition. (**B**) *Firmicutes* relative abundance. (**C**) *Bacteroidota* relative abundance. (**D**) *Verrucomicrobiota* relative abundance. CON (control diet); Bac (bacteriophage diet); ETEC (control diet with ETEC infection); ETEC + Bac (bacteriophage diet with ETEC infection). *n* = 9 or 11. No colonic contents were available for sample collection when the mice were sampled, which resulted in *n* = 9 for the control group. The bars represented by distinct letters signify significant statistical differences among the treatment groups (*p* < 0.05).

Figure 8 shows the microbiota composition at the phylum level in the colon contents in the mice in response to the experimental treatments. The mice in the ETEC group exhibited a reduced abundance of *Firmicutes* and *Bacteroidota*, as well as an augmented *Verrucomicrobiota* presence in their colon contents, relative to the CON group (*p* < 0.05). However, in contrast to the ETEC group, the mice from the ETEC + Bac group had increased *Bacteroidota* relative abundance and reduced *Verrucomicrobiota* relative abundance in the colon contents (*p* < 0.05).

**Figure 9:**
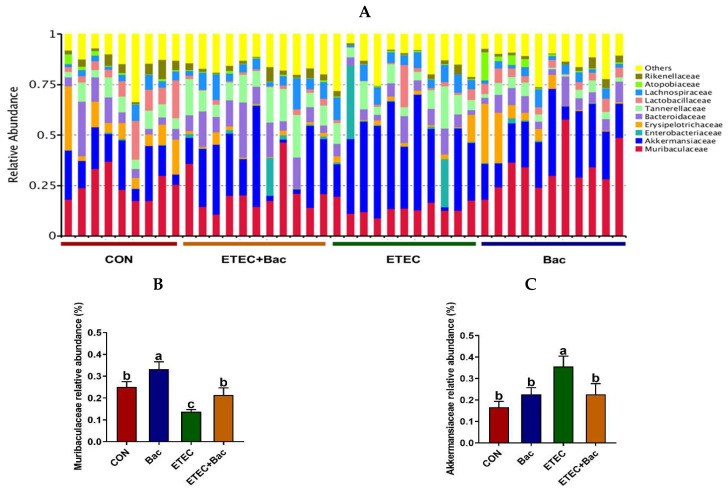
Microbiota composition at the family level (top 10) in the colon contents in the mice in response to the experimental treatments. (**A**) Microbiota composition. (**B**) *Muribaculaceae* relative abundance. (**C**) *Akkermansiaceae* relative abundance. CON (control diet); Bac (bacteriophage diet); ETEC (control diet with ETEC infection); ETEC + Bac (bacteriophage diet with ETEC infection). *n* = 9 or 11. No colonic contents were available for sample collection when the mice were sampled, which resulted in *n* = 9 for the control group. The bars represented by distinct letters signify significant statistical differences among the treatment groups (*p* < 0.05).

The microbiota composition in the colon contents of the mice, at the family level, in response to the experimental treatments is illustrated in Figure 9. Compared with the CON group, the mice from the ETEC group had decreased *Muribaculaceae* relative abundance and increased *Akkermansiaceae* relative abundance in the colon contents (*p* < 0.05). However, in contrast to the ETEC group, the mice belonging to the ETEC + Bac group exhibited a higher *Muribaculaceae* relative abundance, while experiencing a decline in *Akkermansiaceae* relative abundance in the colon contents (*p* < 0.05).

**Figure 10:**
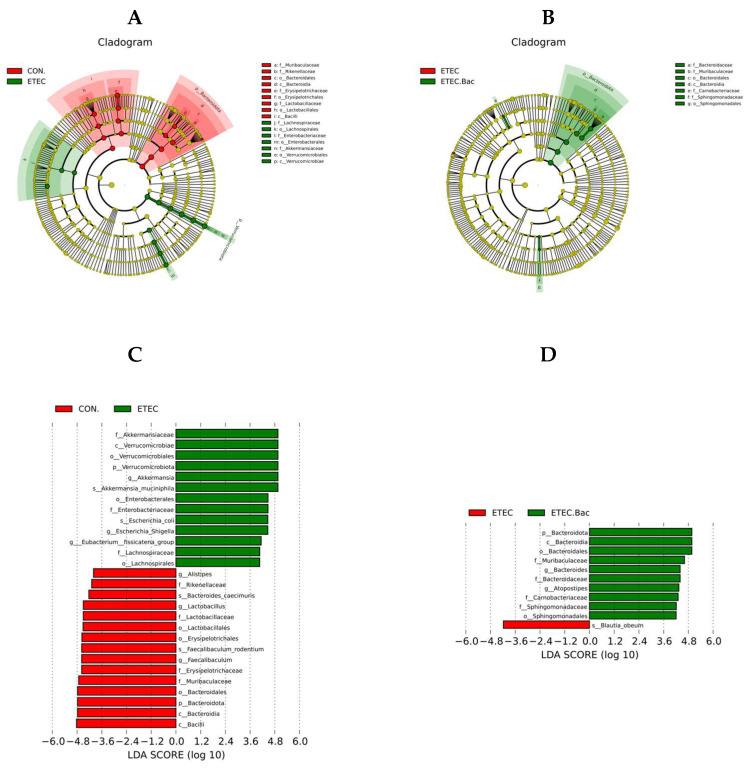
The LEfSe analysis of gut microbiota in the mice from the experimental treatments. (**A**) Cladogram showing the phylogenetic relationships of bacteria taxa between the CON and the ETEC group. (**B**) Cladogram showing the phylogenetic relationships of bacteria taxa between the ETEC and the ETEC + Bac group. (**C**) The LDA scores between the CON and the ETEC group. (**D**) The LDA scores between the ETEC and the ETEC + Bac group. CON (control diet); Bac (bacteriophage diet); ETEC (control diet with ETEC infection); ETEC.Bac (bacteriophage diet with ETEC infection). *n* = 9 or 11. No colonic contents were available for sample collection when the mice were sampled, which resulted in *n* = 9 for the control group. Differentially expressed taxa with the LDA scores > 4.0.

LEfSe analysis was further performed to identify the specific altered microbiota (Figure 10). Compared with the CON group, the mice from the ETEC group had greater relative abundance of *Akkermansiaceae*, *Verrucomicrobiae*, *Verrucomicrobiales*, *Verrucomicrobiota*, *Akkermansia*, *Akkermansia muciniphila*, *Enterobacterales*, *Enterobacteriaceae*, *Escherichia coli*, *Escherichia Shigella*, *Eubacterium fissicatena* group, *Lachnospiraceae*, and *Lachnospirales*, and lower relative abundance of *Bacilli*, *Bacteroidia*, *Bacteroidota*, *Bacteroidales*, *Muribaculaceae*, *Erysipelotrichaceae*, *Faecalibaculum*, *Faecalibaculum rodentium*, *Erysipelotrichales*, *Lactobacillales*, *Lactobacillaceae*, *Lactobacillus*, *Bacteroides caecimuris*, *Rikenellaceae*, and *Alistipes* in the colon contents (*p* < 0.05). Compared to the ETEC group, the mice from the ETEC + Bac group had elevated relative abundance of *Bacteroidota*, *Bacteroidia*, *Bacteroidales*, *Muribaculaceae*, *Bacteroides*, *Bacteroidaceae*, *Atopostipes*, *Carnobacteriaceae*, *Sphingomonadaceae*, and *Sphingomonadales*, and lower *Blautia obeum* relative abundance in the colon contents (*p* < 0.05).

### 2.8. Spearman’s Correlations Analysis

**Figure 11:**
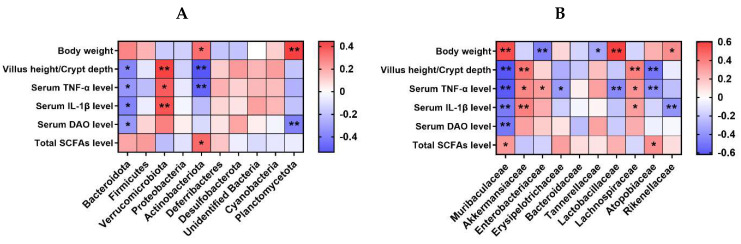
Spearman’s correlation analysis for the selected parameters and gut microbiota at the phylum (**A**) or family (**B**) level (top 10) in the colon content of the mice. The color blue indicates a negative correlation, whereas red shows a positive correlation. *n* = 9 or 11. No colonic contents were available for sample collection when the mice were sampled, which resulted in *n* = 9 for the control group. * *p* < 0.05, ** *p* < 0.01.

Figure 11 exhibits Spearman’s correlation analysis for the selected parameters and microbiota relative abundance at the phylum and family level (top 10) in the colon contents of the mice. At the phylum level, there was a negative correlation between the abundance of *Bacteroidota* and variables such as VH/CD, as well as the serum levels of TNF-α, IL-1β, and DAO (*p* < 0.05). The relative abundance of *Verrucomicrobiota* showed a positive correlation with VH/CD, along with the serum levels of TNF-α and IL-1β (*p* < 0.05). The relative abundance of *Actinobacteriota* was positively correlated with body weight and total SCFA level in caecum contents, while it was negatively correlated with VH/CD and serum TNF-α level (*p* < 0.05). The abundance of *Planctomycetota* exhibited a positive correlation with body weight while displaying a negative correlation with the serum DAO level (*p* < 0.05).

At the family level, the abundance of *Muribaculaceae* was positively correlated with body weight and total SCFA level in caecum contents, while it was negatively correlated with VH/CD and serum TNF-α, IL-1β, and DAO levels (*p* < 0.05). There was a positive correlation between the relative abundance of *Akkermansiacea* and VH/CD, as well as serum TNF-α and IL-1β levels (*p* < 0.05). *Enterobacteriaceae* relative abundance was positively correlated with serum TNF-α level, while it was negatively correlated with body weight (*p* < 0.05). The relative abundance of *Erysipelotrichaceae* showed a negative correlation with the serum TNF-α level (*p* < 0.05). Likewise, the relative abundance of *Tannerellaceae* displayed a negative correlation with body weight (*p* < 0.05). *Lactobacillaceae* relative abundance was positively correlated with body weight, while it was negatively correlated with serum TNF-α level (*p* < 0.05). The relative abundance of *Lachnospiraceae* showed a positive correlation with the VH/CD and the serum levels of TNF-α and IL-1β (*p* < 0.05). *Atopobiaceae* relative abundance was positively correlated with total SCFA level, while it was negatively correlated with VH/CD and serum TNF-α level (*p* < 0.05). The relative abundance of *Rikenellaceae* demonstrated a positive correlation with body weight, whereas it exhibited a negative correlation with the serum level of IL-1β (*p* < 0.05).

## 3. Discussion

The primary objective of this study was to investigate the protective effects of bacteriophage administration on diarrhea and intestinal impairment induced by ETEC, along with its potential underlying mechanism, in a newly weaned mouse model. In this study, there was a decrease in the body weight and an increase in the diarrhea score observed in the mice infected with ETEC at 24 h post-infection. The symptoms observed in this study align with the findings of Chen et al. (2023) and Wang et al. (2022) [20,21], confirming the successful establishment of the ETEC infection model. Importantly, compared with the ETEC group, the mice from the ETEC + Bac group exhibited increased body weight and decreased diarrhea score at 48 h post-infection of ETEC. Similarly, previous studies have shown that dietary or oral administration with bacteriophage has effectively alleviated ETEC infection symptoms in weaned piglets [12,13]. These results support our hypothesis that bacteriophage could counteract the decrease in body weight and occurrence of diarrhea, which are the primary symptoms of ETEC infection.

The findings of the intestinal histomorphology analysis support the alteration of the body weight diarrhea phenotype. The intestinal tract serves as the primary target organ for ETEC infection [22]. Compared to the CON group, the mice with ETEC infection exhibited impaired jejunal histopathology, as evidenced by an elevation in CD and a reduction in VH as well as the VH/CD proportion. Intestinal histomorphology serves as a crucial indicator of intestinal function and overall health [23]. However, the bacteriophage administration effectively alleviated the deleterious consequences of ETEC infection on jejunal histopathology in the mice, as suggested by the reduced CD and the increased VH and VH/CD in the mice from ETEC + Bac group vs. ETEC group. The results suggest that bacteriophage administration effectively alleviates the impairment of intestinal histomorphology caused by ETEC infection.

The intestinal barrier serves as a crucial protective barrier against pathogens and toxins, and heightened intestinal permeability is associated with the occurrence of intestinal damage [24]. The mice in the ETEC group displayed compromised intestinal barrier function when compared to the CON group. This was demonstrated by an elevation in serum DAO levels, which is an indicator of intestinal barrier integrity [25]. The tight junction serves as a vital structure in upholding intestinal barrier integrity and permeability, with its essential functional components being tight junction proteins, such as Claudin-1, ZO-1, and Occludin [26,27]. In this study, the mRNA and protein levels of Claudin-1, ZO-1, and Occludin were observed to be down-regulated in the jejunum mucosa of the mice from the ETEC group compared to the CON group. The findings regarding tight junction proteins have provided additional evidence to support the assertion that the ETEC infection has led to a compromised integrity of the intestinal barrier [28]. Importantly, the bacteriophage administration enhanced the intestinal barrier function of the ETEC-infected mice. This is evident from the reduction in serum DAO level and the increased mRNA and protein levels of Claudin-1, ZO-1, and Occludin in the jejunum mucosa of the mice from the ETEC + Bac group vs. the ETEC group. The findings indicate that the dietary administration of bacteriophage effectively mitigates the impairment of intestinal barrier function caused by ETEC infection.

Previous research have demonstrated that intestinal barrier dysfunction caused by diarrhea is usually accompanied by an inflammatory response [29,30]. We assessed the levels of TNF-α and IL-1β in the mice’s serum. The serum levels of TNF-α and IL-1β exhibited a notable increase in the mice from the ETEC group compared to the CON group, thus indicating that the ETEC infection led to systemic inflammation [31]. This outcome aligns with the findings observed in a weaned pig model [32]. Simultaneously, in the current experiment, it was observed that intestinal inflammation occurred in conjunction with the TLR-4/NF-κB pathway activation in response to ETEC infection. However, dietary bacteriophage administration decreased both systemic and intestinal inflammation. Specifically, the serum TNF-α and IL-1β concentrations were decreased in the mice from the ETEC + Bac group vs. the ETEC group. In contrast to the ETEC group, the mice from the ETEC + Bac group exhibited the down-regulation of mRNA levels for *TLR-4* and *NF-κB-p65*, as well as down-regulated protein levels for TLR-4, NF-κB-p65, and p-NF-κB-p65. Furthermore, the mRNA levels of *TNF-α* and *IL-6* were observed to be down-regulated in the jejunal mucosa of the mice belonging to the ETEC + Bac group in comparison to the ETEC group. The Toll-like receptor 4 (TLR-4) is recognized for its specificity in recognizing the lipopolysaccharide in Gram-negative pathogenic bacteria, including *Escherichia coli* [33]. The TLR-4 further triggers NF-κB pathway activation upon the recognition of lipopolysaccharide, thereby inducing the expression of subsequent inflammatory factors [34]. This could potentially clarify the findings of our study, suggesting that the administration of bacteriophage has mitigated intestinal inflammation via regulating the TLR-4/NF-κB pathway.

The SCFAs are widely recognized for their role as significant sources of energy for intestinal cells, contributing to the maintenance of intestinal health and providing animals with energy [35]. Moreover, SCFAs have been found to play a vital role in the regulation of intestinal immunity, inflammation, and metabolism [36]. The production of SCFAs is tightly controlled through dietary interventions [37]. Compared with the CON group, the mice with ETEC infection exhibited reduced levels of acetic acid, propionic acid, butyric acid, isobutyric acid, and total SCFAs in the caecum contents. However, the levels of acetic acid, propionic acid, butyric acid, and total SCFAs in the caecum content were elevated in the mice from the ETEC + Bac group vs. the ETEC group. This finding suggests that the administration of bacteriophages has a positive impact on the production of intestinal SCFAs in mice infected with ETEC.

The gut microbiota is a well-established and intricately balanced ecosystem that performs a diverse array of advantageous functions for the host, notably safeguarding against invasion by pathogens [38]. It is widely acknowledged that pathogenic infections in the intestine have an impact on the diversity and composition of gut microbiota [39]. The current study demonstrates a decrease in microbiota diversity in the ETEC-infected mice, as indicated by a reduction in the Shannon index within the colon contents. This is in accordance with the findings of Chen et al. (2024) [4]. Additionally, the microbiota composition was influenced by the ETEC infection. Specifically, the mice from the ETEC group exhibited a decrease in the abundance of *Firmicutes*, *Bacteroidota*, and *Muribaculaceae*, as well as an elevation in the abundance of *Verrucomicrobiota* and *Akkermansiaceae* present in the colon contents. Similarly, Wang et al. (2022) observed that ETEC infection resulted in a decrease in the *Firmicutes* abundance and the *Firmicutes*/*Bacteroidetes* ratio in the fecal samples of mice [21]. However, compared with the ETEC group, the mice from the ETEC + Bac group had increased Shannon index in the colon contents. Bao et al. (2018) also found that bacteriophage administration elevated the alpha diversity in the microbiota present in the feces of C57BL/6 mice [40]. In the present study, the mice from the ETEC + Bac group had elevated relative abundance of *Bacteroidota* and *Muribaculaceae* and decreased relative abundance of *Verrucomicrobiota* and *Akkermansiaceae* in the colon contents. These findings indicate that the gut microbiota’s diversity and composition could be regulated by bacteriophage treatments, as similarly stated by Bao et al. (2018) [40].

Spearman’s correlation analysis was performed for the selected parameters and microbiota relative abundance at the phylum and family level (top 10) in the colon contents of the mice. At the phylum level, there was a negative correlation observed between the abundance of *Bacteroidota* and the measurements of VH/CD, and the serum levels of TNF-α, IL-1β, and DAO. *Verrucomicrobiota* relative abundance was positively correlated with VH/CD, and serum TNF-α and IL-1β levels. At the family level, *Muribaculaceae* relative abundance was positively correlated with the body weight and total SCFA level in the caecum contents, while it was negatively correlated with VH/CD, and the serum levels of TNF-α, IL-1β, and DAO. The abundance of *Akkermansiaceae* exhibited a positive correlation with VH/CD, and serum TNF-α and IL-1β levels. Collectively, the protective effects of bacteriophage on ETEC-induced intestinal impairment, inflammation, and intestinal barrier function are associated with regulating the abundance of *Bacteroidota* and *Muribaculaceae* in the colon contents of mice.

To investigate the possible effects of administering bacteriophages to healthy mice without ETEC infection, a comparison was conducted between two groups: the Bac group and the CON group. The results indicate that the bacteriophage administration did not have any negative effects on the healthy mice based on the observations of body weight and diarrhea phenotype, intestinal histomorphology, intestinal barrier function, inflammatory response, and SCFA levels. Regarding gut microbiota, the administration of bacteriophage had a positive impact, as indicated by an increase in the diversity of gut microbiota and the abundance of *Muribaculaceae* in the healthy mice without ETEC infection.

Last but not least, an intriguing question arises: why do the additive effects of the bacteriophages used appear to be similar to those observed in many studies conducted on *E. coli* diarrhea in animals through the utilization of probiotics? This phenomenon could be explained by the similarities and differences between bacteriophages and probiotics. Bacteriophages are naturally occurring bacterial predators, rendering them viable for the treatment of bacterial infections [41]. Bacteriophages possess the capability of eliminating pathogenic bacteria and afford the opportunity for probiotics to proliferate in a comparatively less competitive milieu [42]. In contrast, probiotics can potentially facilitate the growth of specific microbes while inhibiting the growth of others, particularly enterobacteria like *Salmonella* and *E. coli*. This effect is primarily attributed to the pH decrease resulting from anaerobic fermentation within the intestine [42].

## 4. Materials and Methods

### 4.1. Animals and Experimental Design

The study protocols were granted approval by the Animal Care and Use Committee of Jiangxi Agricultural University (approval number: JXAUA01; approval date: July, 2021). Forty-four newly weaned male C57BL/6 mice, aged three weeks, were selected for the study. Following a three-day adaptation period, the mice were assigned into four treatment groups, consisting of eleven mice in each respective group. The four treatment groups included (1) the CON group (control diet), (2) the Bac group (bacteriophage diet), (3) the ETEC group (control diet with ETEC infection), and (4) the ETEC + Bac group (bacteriophage diet with ETEC infection). The mice were fed a control diet or 0.2% bacteriophage-supplemented diet for 16 days. The mice were kept in IVC cage systems, and the bedding was replaced once a week [3]. The basal diet was the mouse AIN-93G diet, serving as the control diet. To create the bacteriophage-supplemented diet, corn starch was replaced with a 0.2% bacteriophage cocktail in the basal diet. The bacteriophage utilized is a bacteriophage cocktail that consists of several purified individual bacteriophages specifically targeting *Escherichia coli* strains K88, K99, F18, F41, 987P, O78, and others, with corn starch being utilized as the carrier substance [11]. The concentration of each bacteriophage in the mixture was 10^8^ plaque-forming units per gram (pfu/g). On the 14th day, the mice were administered intraperitoneal injections of either 0.1 mL of phosphate-buffered saline (PBS) or ETEC solution, with a concentration of 1 × 10^7^ CFU/mL per 10 g of body weight. The ETEC strain (specific serotype: O149:K91:K88ac; serial number: CVCC225) utilized in this research was obtained from the Chinese Veterinary Medicine Collection Center. The preparation of the ETEC solution was carried out in adherence to the methodology in our previous study [3].

### 4.2. Data Record and Sample Collection

#### 4.2.1. Growth and Diarrhea Data Record

The mice’s body weight was recorded on days 1, 7, 14, 15, and 16 of the study. Furthermore, their feed intake was recorded to determine the ADFI. The diarrhea scores of the mice were evaluated on days 14, 15, and 16 of the experiment (days 0, 1, and 2 post-infection of ETEC). The diarrhea symptoms in the mice were scored based on the predefined diarrhea score criteria [43].

#### 4.2.2. Serum Sample Collection

Upon the completion of the study, all the mice were sampled for fresh blood by cardiac puncture. The collected blood samples were placed into Eppendorf tubes and tilted at 45° for 20~30 min. And then, the serum was obtained via centrifugation at 3000 rpm for 15 min, and transferred to 200 μL sterile, enzyme-free Eppendorf tubes and stored at −80 °C for further analysis.

#### 4.2.3. Intestinal Tissue Sample Collection

A section from the middle segment of fresh jejunum tissue was washed with sterile saline and the saline attached to the tissue was removed with filter paper and soaked in 4% paraformaldehyde solution for further histomorphology analysis. The rest of the fresh jejunum tissues were subsequently collected for the purpose of sampling jejunal mucosa. The acquired samples of jejunal mucosa were carefully placed into sterile cryopreservation tubes and stored at a temperature of −80 °C until further analysis.

#### 4.2.4. Intestinal Content Sample Collection

The digesta from the colon and caecum were sampled and conserved in liquid nitrogen until the gut microbiota and SCFA analysis.

### 4.3. Laboratory Analysis

#### 4.3.1. Intestinal Histomorphology

The jejunum histomorphology was evaluated following the methodology in our previous study [4]. In short, the jejunum samples were initially fixed in a 4% paraformaldehyde solution, followed by a dehydration process. The samples were then embedded in paraffin and subsequently sectioned for staining with hematoxylin and eosin. The stained sections were captured using an EVOS microscope (Advanced Microscopy Group, Bothell, WA, USA). A minimum of four images were captured for every tissue section, and the measurements of jejunal VH and CD were conducted using Image-Pro Plus 6.0 (Media Cybernetics, Rockville, MD, USA).

#### 4.3.2. Serum Parameters

The serum parameters, including DAO, TNF-α, IL-1β, and IL-6 levels, were measured using a mouse ELISA Kit (Shanghai Enzyme-linked Biotechnology Co., Ltd., Shanghai, China). The analysis procedure was carried out in accordance with the instructions provided by the manufacturer.

#### 4.3.3. Real-Time Quantitative PCR (RT-qPCR)

The RT-qPCR was performed utilizing the methods described in our recent study [4]. In brief, the total RNA was extracted from the jejunum mucosa using TRIzol reagent (TransGen Biotech, Beijing, China), followed by the assessment of both the quality and quantity of the extracted RNA. Thereafter, the RNA was subsequently reverse-transcribed into cDNA utilizing a commercially available cDNA synthesis kit (TransGen Biotech, Beijing, China). The RT-qPCR was conducted on the CFX96 RT-PCR Detection System, manufactured by Bio-Rad (Hercules, CA, USA), utilizing an SYBR Green kit (TransGen Biotech, Beijing, China). The details of the RT-qPCR primers are presented in Table 1. The 2^−ΔΔCT^ method was employed to determine the relative mRNA levels of genes [44], with 18S ribosomal RNA (18S rRNA) as the internal reference.

#### 4.3.4. Western Blotting

The extraction of total protein from the jejunum mucosa was carried out utilizing a commercial kit procured from Solarbio, based in Beijing, China. The protein concentrations were measured using a BCA kit (TransGen Biotech, Beijing, China). The Western blotting procedure was conducted in accordance with our prior study [45]. The primary antibodies utilized were as follows: anti-β-actin antibody, anti-Claudin-1 antibody, anti-Occludin antibody, anti-ZO-1 antibody, anti-TLR-4 antibody, anti-NF-κB p65 antibody, and anti-p-NF-κB p65 antibody (at a dilution ratio of 1:1000, Cell Signaling, Danver, MA, USA). The signal was detected utilizing an enhanced chemiluminescence system and quantified through the utilization of the ImageJ 1.53t software (NIH, MA, USA).

#### 4.3.5. Gut Microbiota Analysis

The total genomic DNA was extracted from colon digesta using a commercially available kit (Qiagen, Duesseldorf, Germany). The bacterial 16S rDNA was amplified by the primers as follows: 515F 5′-GTGCCAGCMGCCGCGGTAA-3′ and 806R 5′-GGACTACHVGGGTWTCTAAT-3′ in the PCR system. The PCR products were purified using the Qiagen Gel Extraction Kit, produced by Qiagen in Germany. The sequencing and bioinformatics analysis was carried out on the Illumina NovaSeq platform of NovoGene (Beijing, China). The high-quality sequences with ≥97% similarity were allocated to operational taxonomic units (OTUs) at a similarity level of 97% using the UPARSE software, and each OTU was annotated with the Silva Database. The alpha diversity (Chao 1 index and Shannon index) was obtained by QIIME 1.7.0. Unweighted principal component analysis was utilized to objectively quantify the compositional dissimilarities observed within the microbial communities based on OTUs. To determine the significant variations in the beta-diversity of microbiota among the treatments, a PERMANOVA was conducted using the Adonis procedure in order to calculate *p* values. Linear discriminant analysis effect size was used to identify significant differences in the abundance of microbial taxa between the groups followed by linear discriminant analysis to measure the effect size of each abundant taxon. Spearman’s correlation analysis was conducted to examine the correlation between the gut microbiota and selected parameters.

#### 4.3.6. Determination of SCFA Concentrations

The concentrations of SCFAs in cecal digesta were determined using gas chromatography [4]. Briefly, approximately 100 mg digesta samples were weighed followed by SCFA extraction. The cecal digesta was suspended in a solution of water containing 25% metaphosphoric acid and subsequently homogenized using a vortex for approximately 2 min. The suspension was then incubated at a temperature of 4 °C for a duration of 30 min, following which it was subjected to centrifugation at a speed of 12,000× *g* for 10 min at 4 °C in order to obtain the supernatants. And then, the SCFAs were measured using a gas chromatograph system (GC-2014, Shimadzu Corporation, Kyoto, Japan). The system program follows the subsequent procedure: Firstly, it maintains a temperature of 110 °C for 30 s. Subsequently, the temperature steadily increases at a rate of 10 °C per minute until reaching 120 °C. Following this, the system maintains a temperature of 120 °C for 4 min. Finally, the temperature gradually rises to 150 °C over a period of 3 min. Finally, the concentration of each SCFA was quantified using the standard curve established from six different concentrations.

### 4.4. Statistical Analysis

Statistical analysis was performed by utilizing the SPSS 21.0 software (SPSS, Chicago, IL, USA). An one-way analysis of variance was conducted, and then the Duncan post hoc test was employed to assess differences among the treatments. Statistical significance was defined as a *p* value of less than 0.05.

## 5. Conclusions

It is concluded that the administration of bacteriophages alleviated diarrhea and intestinal impairment induced by ETEC in the newly weaned mice by regulating intestinal inflammation and microbiota. This finding indicates the significant potential of bacteriophages in treating and preventing diseases caused by pathogens.

## Figures and Tables

**Table 1:** Primer sequences used for RT-qPCR.

Gene	Primer Sequence (5′-3′)		Product Size (bp)	Accession Number
*MCP-1*	Forward Reverse	ATTGGGATCATCTTGCTGGT CCTGCTGTTCACAGTTGCC	108	NM_011333.3
*ZO-1*	Forward Reverse	GATCCCTGTAAGTCACCCAGA CTCCCTGCTTGCACTCCTATC	154	NM_009386.2
*Occludin*	Forward Reverse	TTGAAAGTCCACCTCCTTACAGA CCGGATAAAAAGAGTACGCTGG	129	NM_001360538.1
*Claudin-1*	Forward Reverse	GGGGACAACATCGTGACCG AGGAGTCGAAGACTTTGCACT	100	NM_016674.4
*NF-KB-p65*	Forward Reverse	AGACCCAGGAGTGTTCACAGACC CACCAGGCGAGTTATAGCTTCAGG	139	XM_006531694.4
*TLR-4*	Forward Reverse	TTCAGAACTTCAGTGGCTGGATT CCATGCCTTGTCTTCAATTGTTT	64	NM_021297.3
*IL-1β*	Forward Reverse	TCGCTCAGGGTCACAAGAAA CATCAGAGGCAAGGAGGAAAC	73	XM_006498795.3
*IL-6*	Forward Reverse	GAGGATACCACTCCCAACAGACC AAGTGCATCATCGTTGTTCATACA	141	NM_001314054.1
*TNF-α*	Forward Reverse	TGGGACAGTGACCTGGACTGT TTCGGAAAGCCCATTTGAGT	67	NM_001278601.1
*18S rRNA*	Forward Reverse	GTAACCCGTTGAACCCCATT CCATCCAATCGGTAGTAGCG	151	NR_046233.2

## Data Availability

Data will be made available upon request.
